# Risk factors for *Leptospira* seropositivity in rural Northern Germany, 2019

**DOI:** 10.1017/S0950268822001972

**Published:** 2022-12-27

**Authors:** Saskia Schmitz, Christina Princk, Kristin Meyer-Schlinkmann, Maren Mylius, Nadja S. Bier, Armin Baillot, Masyar Monazahian, Rainer G. Ulrich, Anne Mayer-Scholl, Johannes Dreesman

**Affiliations:** 1 Public Health Agency of Lower Saxony, Hanover, Germany; 2 Department Biological Safety, German Federal Institute for Risk Assessment, Berlin, Germany; 3 Institute of Novel and Emerging Infectious Diseases, Friedrich-Loeffler-Institut, Greifswald-Insel Riems, Germany; 4 German Centre for Infection Research (DZIF), partner site Hamburg-Lübeck-Borstel-Riems, Greifswald-Insel Riems, Germany

**Keywords:** *Leptospira* spp, risk factors, rodent-borne diseases, seroprevalence

## Abstract

We investigated seroprevalence and factors associated with *Leptospira* spp. infections in humans in rural Northern Germany. Sera of 450 participants were tested for leptospira-reactive IgG antibodies by two enzyme-linked immunosorbent assays (ELISA). A narrow (specific) and a broad (sensitive) case definition were applied and results compared in the analysis. Personal data were collected via questionnaire and associations with the serostatus were investigated by multivariable logistic regression. The seroprevalence estimates were 1.6% (95%-confidence interval (CI) = 0.63–3.2) under the narrow and 4.2% (95%-CI = 2.6–6.5%) under the broad case definition. Few (14%) participants knew about the pathogen. No seropositive participant recalled a prior leptospirosis diagnosis. Spending more than two hours a week in the forest was significantly associated with anti-leptospira IgG in both models (broad case definition: adjusted odds ratio (aOR) = 2.8, 95%-CI = 1.2–9.1; narrow case definition: aOR = 11.1, 95%-CI = 1.3–97.1). Regular cleaning of storage rooms was negatively associated in the broad (aOR = 0.17, 95%-CI = 0.03–0.98) and touching a dead rodent in the past 10 years in the narrow case definition model (aOR = 0.23, 95%-CI = 0.05–1.04). Our findings support risk factors identified in previous investigations. To counter the low awareness for the pathogen, we recommend that health authorities communicate risks and preventive measures to the public by using target-group specific channels.

## Introduction

In Germany, the laboratory confirmation of an acute infection with human pathogenic *Leptospira* spp. is notifiable under the Protection against Infection Act of 2001. Since 2001, the annual notification incidence was low but consistent in Germany, ranging between 0.04 and 0.2 cases per 100 000 inhabitants [1].

Leptospirosis is a zoonotic disease that occurs globally and is caused by pathogenic bacteria of the genus *Leptospira,* currently consisting of 64 species and over 260 serovars [2, 3]. In Germany, *Leptospira kirschneri* serovar Grippotyphosa is among the most relevant for human infection [4, 5]. The most important reservoir animals for human infections are rodents, but a range of pets, including dogs and cats, and farm animals can also get infected and shed the pathogen [5]. For cattle, pigs and dogs vaccines against *Leptospira* serovars occurring in Germany are available [5]. For cats, for which studies in Germany described a seroprevalence of 16.0–17.9% [6, 7], no vaccine is approved [5]. Humans are an accidental host for the bacteria [8], which can enter the human body through skin abrasion and the mucosa, typically during contact with contaminated soil or water [5]. Infections in the main reservoirs mostly take an asymptomatic course but become chronic, so that bacteria are excreted lifelong. Human infections typically manifest subclinically or with mild flu-like and unspecific symptoms [8, 9] but can also lead to severe forms with multiple organ failure. Weil´s disease denotes one serious course of the disease, which is characterised by jaundice, acute renal failure and haemorrhages [8, 10].

To date, human vaccines against *Leptospira* spp. are not available in most European countries, including Germany [11]. Following the One Health approach interdisciplinary research groups aim to better learn about the interplay between the environment and the infection rate in rodents to understand what that means for human risk of infection [12]. So far these approaches do not lead to a way to reduce this threat. The knowledge of risk factors and effective protective behaviour, including measures of rodent control, is the most important way to prevent human infections [9]. Occupational exposure has been described for several, mainly outdoor focused, occupational groups like agricultural or sewer workers [4, 8]. However, the awareness of exposures in residential settings or through recreational activities, such as gardening and water sports have risen in recent decades as they constitute a risk of infection for the general public [13–15].

The aim of this study is to assess the seroprevelance of anti-leptospira immunoglobulin G (IgG) in adults in a rural region in Lower Saxony, Germany, and to explore the association of the individual serostatus with the participants´ exposures, knowledge and preventive behaviours.

## Methods

### Recruitment and data collection

This seroprevalence study was conducted in a rural area in the district of Osnabrück in Lower Saxony, Germany, where a parallel rodent trapping is running since 2005 [12, 16]. General practitioners in this rural area were asked to participate through one of the general practitioners, who had previously cooperated with study partners in scientific investigations. Two general practitioners took part in this study. From February to September 2019, their patients were invited to participate in the study. After the participants gave their informed consent, a blood sample of eight to ten millilitres was taken and sent by post the same or latest the next day to the laboratory for testing. Blood samples that were stored overnight were kept at fridge temperature. The participants filled out a self-administered paper-based questionnaire, that collected data on socio-demographic characteristics, living conditions, health parameters, exposures to potential risk factors, contact to rodents, control measures for rodents, preventive measures against rodent-borne pathogens, knowledge about leptospirosis, as well as a previous leptospirosis diagnosis. The questionnaires included questions about severe symptoms, such as meningitis, jaundice, kidney injury and conjunctivitis, which occurred without pathogen identification in connection with an acute disease in the past. Selected exposure information was collected separately for weekdays and weekends to aid participant´s recollection of behavioural patterns during leisure time (see Supplement).

Neither the practitioners nor the participants received financial incentives for participation. Participants received the results of the blood sample analysis upon their next consultation with their general practitioner following the completion of the questionnaire.

### Analysis of samples

Blood samples were analysed by a commercial enzyme-linked immunosorbent assay (ELISA) for IgG antibodies against *Leptospira* spp. (SERION ELISA classic Leptospira IgG, Institut Virion\Serion GmbH, Würzburg, Germany) according to manufacturer´s specifications. According to the manufacturer, the test specificity was >99% and the sensitivity 94.7%.

All samples were also tested using an in-house ELISA for IgG antibodies against *Leptospira* spp., which had been validated with human sera from five different populations in Germany using Bayesian modelling [17]. Using sera from people exposed to *Leptospira* spp. and suspected of infection a clinical sensitivity of 83.0% and a clinical specificity of 98.5% were determined. Using sera of people without known exposition to the bacteria a subclinical sensitivity of 85.7% and a subclinical specificity of 99.1% were determined [17].

Samples were classified as positive, negative or intermediate. For the Virion/ Serion ELISA upper and lower cut-off values were calculated according to manufacturer's instructions: blank values were deducted from mean values of provided standard sera and the resulting optical densities were multiplied with a batch-specific numerical value. The classification for the in-house ELISA was conducted as described previously [17].

The presence or absence of antibodies in the individual, as indicated by the ELISAs, is referred to in this text as ‘serostatus’ or ‘seropositivity’ and ‘seronegativity’ respectively. The proportion of participants tested seropositive will be referred to as the study group´s seroprevalence.

### Statistical analysis

Two case definitions were applied. According to the broad case definition (CD), participants were rated seropositive if they tested positive in at least one ELISA. In the narrow CD, participants were regarded seropositive if they tested positive in both ELISAs. Participants that tested negative in both ELISAs (broad CD) or in at least one ELISA (narrow CD) were included in the analysis as seronegative. For both versions the statistical analysis was conducted separately and results were compared.

For the seroprevalences exact 95% confidence intervals (95%-CI) were calculated [18]. Associations between potential risk factors and the serostatus were analysed for all participants who filled out the questionnaire using uni- and multivariable logistic regression. The estimated effect is presented by crude odds ratios (OR) and adjusted odds ratios (aOR). The Wald test and exact 95%-CI were calculated to assess statistical significance [18]. A significant association was assumed if the *p*-value was <0.05 and the CI did not span from below to above one, thereby covering it. Stepwise backward elimination [19] was used to select variables for the multivariable model among all variables with a *p-*value < 0.25 in the univariable analysis. Collinearity of the exposure variables was assessed prior to elimination, using Pearson´s phi coefficient. For pairs of variables that were indicated to be affected by collinearity (*r* > 0.3) either a new combinatory variable was created or only one variable was included in the model as a proxy, representing all collinear variables [20]. Age and sex were included in the multivariable model as potential confounders. In both models confounding and interaction between variables were tested by stratified analysis using Mantel-Haenszel´s and Tarone´s test of homogeneity. Continuous variables that did not meet the assumption of a linear relationship with the logit of the dependent variable were categorised. Exposure variables with ordered categories were treated as continuous in the logistic regression model if they met the assumption of linearity tested via likelihood-ratio test. Their corresponding OR and aOR express the change per category transition.

Associations between unexplained symptoms and serostatus were analysed using Fisher´s exact test. The same method was used to investigate the association between knowledge about the disease and serostatus. *χ*^2^ test was used to analyse the association between relevant exposures and preventive measures. P-values were used to assess statistical significance, with the significance level set at 0.05.

All statistical analysis was conducted using Stata SE15^®^ (StataCorp., USA). Microsoft^®^ Access 2016 (Microsoft, USA) was used as the database system.

### Ethical approval

Ethical approval for the study was obtained from the ethics committee of the medical chamber of Lower Saxony (procedure number Bo/13/2018).

## Results

Sera of 450 participants were tested with both ELISAs. The study population included 232 (51.6%) women. The age range was 18–85 years, with a median of 59 years. The questionnaire was filled out by 449 participants.

Of the 450 samples, 19 tested positive in at least one of the ELISAs, leading to a seroprevalence of 4.2% (95%-CI = 2.6–6.5%) when applying the broad CD, and seven tested positive in both ELISAs, corresponding to a seroprevalence of 1.6% (95%-CI = 0.63–3.2%) in the narrow CD (Table 1). Both tests identified seven participants (1.6%) as seropositive, six (1.3%) as intermediate and 405 (90.2%) as seronegative (Table 1). If viewed separately the Virion/ Serion ELISA yielded a seroprevalence of 2.4% (95%-CI = 1.2–4.3%) and the in-house ELISA a seroprevalence of 3.3% (95%-CI = 1.9–5.4%) (Table 1).

**Table 1.** Results of Virion/ Serion ELISA and in-house ELISA of German reference laboratory for *Leptospira* spp.

In-house ELISA				
Virion/ Serion ELISA	Positive	Intermediate	Negative	Total
Positive	7	3	1	11
Intermediate	0	6	9	15
Negative	8	11	405	424
Total	15	20	415	450

There were no statistical significant associations between seropositivity, according to both CD, a prior diagnosis of leptospirosis, potentially related symptoms and knowledge of the disease and sources of knowledge (Table 2). Around 14% of the participants had knowledge about *Leptospira* spp. or leptospirosis prior to the study (Table 2). Around 23% of participants were aware of press releases about rodent-borne diseases by their local health department. No participant remembered ever having received a diagnosis of *Leptospira* infection or leptospirosis (Table 2).

**Table 2.** Distribution and association of study characteristics with the serostatus of anti-leptospira IgG under the broad and narrow case definition

	Broad case definition^a^						Narrow case definition^b^					
Characteristic	Seropositive		Seronegative		Total	*p*^c^	Seropositive		Seronegative		Total	*p*^c^
	*n* (18)	%	*n* (431)	%	*n* (449)		*n* (7)	%	*n* (442)	%	*n* (449)	
Ever diagnosed with…												
Leptospirosis	0	0.0	0	0.0	0	.	0	0.0	0	0.0	0	.
Meningitis	0	0.0	9	2.1	9	0.64	0	0.0	9	2.0	9	1.00
Conjunctivitis	5	27.8	89	20.6	94	0.64	2	28.6	92	20.8	94	0.57
Jaundice	0	0.0	18	4.2	18	0.62	0	0.0	18	4.1	18	1.00
Kidney dysfunction	1	5.6	22	5.1	23	0.58	0	0.0	23	5.2	23	1.00
Information												
Knowledge of leptospirosis or *Leptospira* spp. prior to study	1	5.6	62	14.4	63	0.49	1	14.3	62	14.0	63	1.00
Aware of press release about rodent-borne diseases of local health department	4	22.2	98	22.7	102	1.00	2	28.6	100	22.6	102	0.66

a Sample is regarded as positive if positive in one or both ELISAs

b Sample is regarded as positive if positive in both ELISAs

c *p*-values based on Fisher's exact test

Among the risk factors, ‘spending leisure time in the forest during the week’ is the only factor significantly associated with an increased seroprevalence of anti-leptospira IgG in both the univariable and multivariable regression analyses with both CDs. It corresponds with a significant aOR of 2.8 (p = 0.04, 95%-CI = 1.04–7.4) in the broad CD model, where it was entered as a representative variable, i.e. proxy variable for ‘hiking, running or walking during the month’, and of 11.1 (p = 0.03, 95%-CI = 1.3–97.2) in the narrow model, where it was entered as a proxy variable representing ‘hiking, running or walking during the month’ as well as ‘spending leisure time in the forest during the weekend’ (Table 3).

**Table 3.** Univariable and multivariable analysis of selected exposure associated with seropositivity for anti-leptospira IgG under the broad and narrow case definition

				Broad Model^a^										Narrow Model^b^							
		*N* All	*n* Pos	Prevalence		Univariable analysis			Multivarible analysis			*N* All	*n* Pos	Prevalence		Univariable analysis			Multivarible analysis		
No		449	18	%	95%-CI	OR	*p*	95%-CI	aOR	*p*	95%-CI	449	7	%	95%-CI	OR	*p*	95%-CI	aOR	*p*	95%-CI
Variables used for general adjustment																					
1a	Age (c.)^c^	Not included														**0.95**	0.03	0.91–0.996	0.97	0.16	0.92–1.01
1b	Age																				
	18–50	118	5	4.2	1.8–9.8	Ref.^d^			Ref.^d^			Not included									
	51–60	137	6	4.4	2.0–9.4	1.0	0.96	0.31–3.5	1.06	0.92	0.31–3.64										
	61–70	110	4	3.6	1.4–9.3	0.85	0.82	0.22–3.3	0.74	0.67	0.19–2.92										
	>70	84	3	3.6	1.2–11	0.84	0.81	0.19–3.6	0.88	0.87	0.20–4.0										
2	Sex																				
	Male	218	8	3.7	1.8–7.2	Ref.^d^			Ref.^d^			218	1	0.46	0.06–3.2	Ref.^d^			Ref.^d^		
	Female	231	10	4.3	2.3–7.9	1.2	0.72	0.46–3.1	1.02	0.97	0.38–2.7	231	6	2.6	1.2–5.7	5.8	0.11	0.69–49	3.2	0.30	0.35–29
Education																					
3	General education school leaving certificate (years)																				
	12/13	138	6	4.3	2.0–9.4	Ref.^d^						138	3	2.2	0.70–6.5	Ref.^d^					
	10	170	7	4.1	2.0–8.4	0.94	0.92	0.31–2.9				170	3	1.8	0.57–5.3	0.81	0.80	0.16–4.1			
	9	127	4	3.1	1.2–8.1	0.72	0.61	0.2–2.6				127	0	.	.	.					
	None or other certificate	14	1	7.1	0.99–3.7	1.70	0.64	0.19–15				14	1	7.1	0.99–37	3.5	0.30	0.01–0.07			
Evidence for rodent infestation (last 10 yrs)																					
4	Any kind of evidence																				
	Rarely (≤5 times)	121	7	5.8	2.8–12	Ref.^d^			*See 9.*^e^			Not included									
	Often (>5x)	328	11	3.4	1.9–6.0	0.57	0.25	0.21–1.5													
5	Finding rodents faeces (c.)^c^					0.68	0.18	0.39–1.2	*See 9.*^e^			Not included									
6	Finding gnaw marks (c.)^c^					0.66	0.15	0.38–1.2	*See 9.*^e^			Not included									
8	Spotting living rodents																				
	Rarely (≤5x)	Not included										214	5	2.3	0.97–5.5	Ref.^d^			*See 9.*^e^		
	Often (>5x)											235	2	0.85	0.21–3.3	0.36	0.22	0.07–1.9			
9	Noticing change in smell																				
	No	241	13	5.4	3.2–9.1	Ref.^d^						241	6	2.5	1.1–5.4	Ref.^d^					
	Yes	208	5	2.4	1.0–5.7	0.43	0.12	0.15–1.2				208	1	0.48	0.07–3.3	0.19	0.13	0.02–1.6			
Exposure to risk factors																					
10	Touched dead rodent(last 10 yrs)																				
	No	306	16	5.2	3.2–8.4	Ref.^d^			Ref.^d^			Not included									
	Yes	143	2	1.4	0.35–5.4	0.26	0.07	0.06–1.1	**0.23**	**0.056**	0.05–1.04										
12	Cleaning storage rooms (last 10yrs)																				
	Rarely (≤5x)	139	9	6.5	3.4–12	Ref.^d^						139	5	3.6	1.5–8.4	Ref.^d^			Ref.^d^		
	Often (>5x)	310	9	2.9	1.5–5.5	0.43	0.08	0.17–1.1				**310**	**2**	**0.64**	**0.16**–**2.6**	**0.17**	**0.04**	**0.03**–**0.91**	**0.17**	**0.048**	**0.03**–**0.99**
13	Bitten by rodent																				
	No	Not included										439	6	1.4	0.61–3.0	Ref.^d^					
	Yes											10	1	10	1.4–47	8.0	0.07	0.87–74			
Freetime activities																					
14	Swimming in inland waters																				
	No	307	10	3.3	1.8–6.0	Ref.^d^						307	3	0.98	0.31–3.0	Ref.^d^					
	Yes	142	8	5.6	2.8–11	1.77	0.24	0.68–4.6				142	4	2.8	1.1–7.3	2.9	0.16	0.65–13			
15	Participating in water sport per month																				
	No	Not included										295	3	1.0	0.33–3.1	Ref.^d^			*See 14.*^e^		
	Yes											154	4	2.6	0.98–6.7	2.6	0.22	0.57–12			
16	Spending time in the forest/ week																				
	Rarely (<1 h/ week)	274	7	2.6	1.2–5.3	Ref.^d^			Ref.^d^			**274**	**1**	**0.36**	**0.05**–**2.6**	**Ref.^d^**			Ref.^d^		
	Often (≥2 h/ week)	175	11	6.3	3.5–11	2.56	0.06	0.97–6.7	**2.8**	**0.04**	**1.04**–**7.40**	**175**	**6**	3.4	**1.5**–**7.4**	**9.7**	0.04	1.2–81	**11**	**0.03**	**1.3**–**97**
17	Being in the forest/ weekend																				
	<2hrs	Not included										309	3	0.97	0.31–3.0	Ref.^d^			*See 16*^e^		
	= >2 h											140	4	2.9	1.1–7.4	3.0	0.15	0.66–14			
18	Hike/ run/ walk per month																				
	Seldom (<1x/ week)	218	6	2.8	1.2–6.0	Ref.^d^			*See 16*^e^			218	1	0.46	0.06–3.2	Ref.^d^			*See 16.*^e^		
	Often (≥1x/week)	231	12	5.2	3.0–8.9	1.94	0.19	0.71–5.3				231	6	2.6	1.2–5.7	5.8	0.11	0.69–49			
Infection prevention and pest control																					
19	Wearing mask while cleaning storage room																				
	No	223	12	5.4	3.1–9.2	Ref.^d^						Not included									
	Yes	226	6	2.7	1.2–5.8	0.48	0.15	0.18–1.3													
20	Using poison as rodent control																				
	No	262	13	5.0	2.9–8.4	Ref.^d^						262	6	2.3	1.0–5.0	Ref.^d^					
	Yes	187	5	2.7	1.1–6.3	0.53	0.23	0.18–1.5				187	1	0.53	0.07–3.7	0.23	0.18	0.03–1.9			
Living conditions																					
21	Rodent as a pet																				
	No	Not included										367	4	1.1	0.41–2.9	Ref.^d^					
	Yes											82	3	3.7	1.2–11	3.5	0.11	0.76–16			

a Broad case defintion applied: sample is regarded as positive if positive in one or both ELISAs

b Narrow case definition applied: sample is regarded as positive if positive in both ELISAs

c Variable treated as continuous

d Reference category

e Variable was excluded due to collinearity. It is represented by the variable of the indicated number.

‘Cleaning storage rooms, like attics or garages, more than five times in the past ten years’ was associated with lower odds for seropositivity than the reference group under both CDs (narrow CD: aOR = 0.17, *p* = 0.038; broad CD: OR = 0.43, *p* = 0.082) but was only included as a significant factor in the narrow CD model. We did not find significant interactions or confounding by preventive measurements in either model but a significant association of ‘cleaning storage rooms’ with ‘using gloves as protection against rodent-borne pathogens’ (*χ*^2^ = 30.8, *p*< 0.001; data not shown in Tables).

The exposure to rodents, as the main reservoir of *Leptospira* spp., was measured with different variables. Participants, who had touched a dead rodent in the past ten years had an aOR of 0.23 for seropositivity in the broad CD model compared to unexposed participants. This variable entered the broad model as borderline significant (*p* = 0.056, 95%-CI = 0.05–1.04), but did not meet the inclusion criteria for the narrow model. We found a significant association between ‘touching a dead rodent in the past 10 years’ and ‘the use of rodent control’ (*χ*^2^ = 8.03, *p* = 0.005), ‘cleaning rodent traps after usage’ (*χ*^2^ = 9.2, *p* = 0.003), ‘washing hands after touching a dead rodent’ (*χ*^2^ = 30.2, *p* < 0.001) and ‘wearing gloves to prevent rodent-borne infections’ (*χ*^2^ = 27.3, *p* < 0.001, data not shown in tables). Several variables about participants noticing evidence for the presence of rodents, like a change of smell, gnaw marks or rodent faeces, without having a direct contact with the animals were likewise associated with lower odds for seropositivity in the exposed compared to the unexposed group in the univariable analysis but did not meet inclusion criteria for either multivariable model.

In contrast to the low OR of these exposures, ‘being bitten by a rodent’ or ‘owning a rodent as a pet’ were both associated with a higher chance of seropositivity in exposed compared to non-exposed participants in the univariable analysis of the narrow cases. The variables did not meet the inclusion criteria for the broad model.

No significant association of sex and education with the serostatus was found. Age entered the broad model as a categorical variable and did not yield a significant result. It entered the narrow model as a continuous variable and showed a significantly decreased OR (OR = 0.95, *p* = 0.032) for seropositivity per additional year of age. The aOR became closer to one and the statistical significance disappeared in the multivariable model (aOR = 0.97, *p* = 0.16) (Table 3).

Participants were questioned if they ever worked in a predominantly outdoor based or known risk occupation such as farmer, shepherd, gardener, forestry worker, sewer worker, garbage collector or hunter. Around 23% (105/449) of participants stated to have ever worked in any of these occupations. We did not find a significant association between occupation and serostatus.

## Discussion

We observed a seroprevalence for leptospira-reactive IgG antibodies between 1.6%, if a narrow and more specific CD is applied, and 4.2% with a broader and more sensitive CD. Due to the diversity in the *Leptospira* genus not every ELISA detects antibodies for all serovars equally well. For example during an leptospirosis outbreak in strawberry pickers in 2007 described by Desai *et al*. [4] a lack of sensitivity for the serovar Grippotyphosa has been noted by the project partner at the German Federal Institute for Risk Assessment for the used Virion/ Serion ELISA. This serovar has been identified as a causative agent in leptospirosis outbreaks among field workers in Germany and its presence was detected in field and common voles in Germany [4, 5, 21]. As there is no adequate confirmation test available for the pathogen in a seroprevalence study, we combined two ELISAs to supplement each other and defined a spectrum in which we can assume that the true seroprevalence and effects [17]. We found larger effect point estimates under the narrow CD, for which we assume fewer occurrence of false positive but more of false negative results as under the broad CD.

Comparable studies on the seroprevalence of *Leptospira* spp. infections in the German general population are rare. A study of 1050 residents of Baden-Wuerttemberg by Brockman *et al*. from 2010, using the same in-house ELISA we utilised in our study, found a seroprevalence of 4.2% [22]. Another study was conducted on employees of German forestry enterprises. In 2015, Jurke *et al*. found an anti-leptospira IgG seroprevalence of 14.2% in 722 participants from forestry enterprises in North Rhine-Westphalia, using the same in-house ELISA [23]. They presented the seroprevalence of 245 occupationally exposed forestry workers and 171 unexposed office workers separately and found a noticeably lower seroprevalence in the former compared to the latter category (10.2% vs. 17.5%) [23]. The results of these two studies cannot be directly compared to the seroprevalences we calculated based on the broad and narrow CDs, but to the results of the individual ELISAs in our study. We found a seroprevalence of 3.3% (95%-CI = 1.9–5.4) based only on the results of the in-house ELISA, which is comparable to the results of Brockmann *et al*. [22] described for another sample group of the German general public. It was significantly smaller than the seroprevalence observed by Jurke *et al*. [23], which was by far larger than in all other comparable studies.

None of our seropositive participants remembered ever having received the diagnosis of a *Leptospira* spp. infection or leptospirosis. One possible explanation is that the participants experienced mild or subclinical symptoms. This explanation would be supported by the lack of a significant association we found between seropositivity and the participant´s history with experiencing symptoms like meningitis or kidney injury, without pathogen identification in connection with an acute disease in the past. This analysis suggests that their undetected *Leptospira* spp. infection was not accompanied with strong symptoms. The other option is, that patients with unspecific symptoms were not tested for *Leptospira* spp., because it is a rare disease in Germany and thus remained undiagnosed.

To inform about locally relevant rodent-borne diseases the health department of the study district releases information mostly via newspapers, radio and its website. However, the majority of participants were unaware of the pathogen and their local health department as provider of this information. The distribution of such information might be most effective during summer and autumn, when the majority of infections occur, or in connection with floodings, heavy rainfall or water sport events [10, 13, 24, 25]. A broad spectrum of channels such as the traditional media, social media and health care providers should be explored, to reach a wider audience.

We did not find a significant association of sex or occupation with serostatus in our study. In contrast, most notified cases of leptospirosis in Germany are working aged men [1]. The sex difference is usually explained by men being more likely to work in outdoor-occupations that are connected with higher rodent exposure, such as soldiers, construction, agricultural, abattoir and sewer workers or fishers [8]. However, with the increasing importance of recreational exposures and exposures around the house, infections are more likely to affect a broader range of people, not just specific occupational groups or genders [15, 26]. Additionally, in the wake of a changing climate, an increase in heavy rainfall and flooding events is expected in Western Europe. Such events have already shown to facilitate outbreaks in the past and are likely to lead to an increase of leptospirosis outbreaks in the future, independent of occupational exposures [27]. The lack of connection between sex and seropositivity is also shown by Brockmann *et al*. and Jurke *et al*., that do not observe a significant association between those factors neither [22, 23]. The role of occupational exposure in Germany is less clear. Jurke *et al*. describe a higher seroprevalence for occupationally unexposed office workers than occupationally exposed forestry workers, suggesting that leisure time exposures were more relevant for the serostatus than work related exposure [23]. In either case it is likely that workers in established risk occupations, are more likely to be tested for and diagnosed with leptospirosis when experiencing symptoms than the general public and are thus influencing the notified incidence in Germany.

We described several factors associated with seropositivity. Models with both CDs identified ‘leisure time spent in the forest’ being related to higher OR for seropositivity, a result backed by the literature [23]. The forest is a natural habitat for many rodent species and thus exposure to contaminated soils or water is more likely in that surrounding [5]. Furthermore, a recent study detected *Leptospira* spp. in 4.3% of bank voles collected at the study area and 7.5% along a longer transect between North Rhine-Westphalia and Lower Saxony [12].

Surprisingly, we found a reduced chance of seropositivity in participants that reported exposures such as ‘touching a dead rodent’ in the broad CD model and ‘frequently cleaning storage rooms’ compared to unexposed participants in the narrow CD model. For both exposures we found significant associations to preventive behaviours such as ‘wearing gloves to prevent rodent-borne infections’ and ‘washing hands after contact with a rodent’. This connection suggests that participants used protective gear when experiencing those exposures more frequently, thus reducing the risk of infection. Similarly, ‘touching a dead rodent’ showed an association with ‘using pest control measurements’, which suggests that it might have occurred when preventing or countering rodent inhabitation of human spaces. Cleaning a storage room frequently, similarly, helps prevent rodents from settling in it. These associations need to be viewed with some caution, because they present an additional analysis our study was not primarily designed for. However, the results support the notion that keeping the home free of rodents is important to reduce the risk of infection. To prevent infection during maintenance and cleaning work at home, recommended hygienic practices like wearing protective gear should be employed.

Our study included a small subsample of seropositive participants, which determines the low precision of the effects' point estimates, represented by the broad CI in both models. The OR should therefore be consulted for the direction of association and does not provide a precise description of its strength. Additionally, some relevant factors are likely not to show statistical significance unless their OR are sufficiently large. One example of this is the lack of a statistically significant association between seropositivity and water sports, that has been well described in other studies [15, 22] and is seen in the number of outbreaks in connection to water sport events from all over the world [13, 26, 28–30].

This study had certain limitations. The study population was not chosen representative for the general population and might vary from it in several factors like age, gender or education. However, based on national surveillance data no risk area for leptospirosis is known in Germany, and the annual notification incidence during the past 20 years in the selected study area did not differ substantially from other regions in Germany [1]. Therefore, the seroprevalence estimate we observed in this study can give an indication of the seroprevalence in other populations with comparable age and gender structure in a rural area, without a history of large outbreaks.

The retrospective cross-sectional design may have introduced a recall bias in questions on exposures to rodents in the past ten years. It can be assumed, however, that these exposures were not fully forgotten by participants and the bias might apply more to scale than to the overall occurrence.

## Conclusion

In the past two decades several leptospirosis outbreaks occurred in Germany both in occupational and recreational settings [13, 24, 25]. While remaining a rare disease in Germany, further outbreaks are likely and are not limited to occupational settings.

Considering that the route of infection for humans does not seem to depend on the leptospira serovar [8, 10], the risk factors we described based on findings from a small German region can be used to inform health protection efforts in other regions and countries, independent of the dominant leptospira strain. Spending leisure time in the forest was the major factor connected to infections in our study. Considering the health and well-being benefits of this behaviour, the forest should not be avoided [31]. Avoiding contact with water and soil might lower the infection risk in this setting but can also severely limit the quality of the experience. Risks and benefits should be regularly considered and recommendations for preventive behaviour adapted if necessary.

We found that regularly cleaning storage rooms and disposing of dead rodents with proper protective gear can lower the chance for infection. It is important, however, to adhere to hygiene rules such as covering exposed skin and skin abrasions during these activities and washing of hands and clothes afterwards.

Our study showed a low level of awareness for the pathogen in the population. This stresses the need for local health authorities to supply information about effective protective measurements, using target-appropriate communication channels.

## Data Availability

Data is available by requesting access from the authors.

## Supplementary material

For supplementary material accompanying this paper visit http://doi.org/10.1017/S0950268822001972. click here to view supplementary material
