# Supplementary material for: Risk factors for Leptospira seropositivity in rural Northern Germany, 2019
Source: Epidemiol Infect. 2022 Dec 27;151:e17. doi: 10.1017/S0950268822001972 (PMC9990393; doi:10.1017/S0950268822001972)
Supplement: Supplementary file 1 [file S0950268822001972sup001.pdf]

Teilprojekt 2

ID-Nr.

Befragung von Hausarztpatienten/innen

in Niedersachsen

Im Rahmen eines Verbundprojektes* zur „Verbesserung der Öffentlichen Gesundheit durch ein besseres Verständnis der Epidemiologie nagetierübertragener Krankheiten“

Niedersächsisches Landesgesundheitsamt (NLGA)
Roesebeckstr. 4-6
30449 Hannover

Gefördert vom:
Bundesministerium für Bildung und Forschung

Ihre Ansprechpartnerinnen im NLGA:
Maren Mylius Tel.: 0511-4505-307
Christina Princk Tel.: 0511-4505-268

* Verbundprojekt RoBoPub: Rodent Borne Pathogens and Public Health; deutsche Übersetzung: Nagetierübertragene Erreger und öffentliche Gesundheit

Liebe Studienteilnehmerin, lieber Studienteilnehmer,

Vielen Dank, dass Sie sich für eine Teilnahme an unserer Studie entschieden haben. Sie leisten damit einen wertvollen Beitrag in der Erforschung von Hantavirus-Erkrankungen und Leptospirosen.

Die Beantwortung des Fragebogens wird **ca. 20 Minuten** Ihrer Zeit in Anspruch nehmen. Nähere Informationen zum Ziel der Studie und zum Umgang mit Ihren Daten entnehmen Sie bitte der beiliegenden Studieninformation.

Sollten noch Fragen offengeblieben sein, kontaktieren Sie uns gerne unter den angegebenen Kontaktdaten.

Hinweise zum Ausfüllen des Fragebogens

- Lesen Sie jede Frage vor dem Beantworten sorgfältig durch und markieren Sie dann die Antwort, die am besten auf Sie zutrifft. Kreuzen Sie gegebenenfalls auch „Nein“ oder „Weiß nicht“ als Antwort an, wenn diese Antwort am besten zutrifft.
- In die folgenden Felder tragen Sie bitte Zahlen ein.

Beispiel: **Ausfülldatum des Fragebogens:** |1|4|.|0|6|.|2|0|1|8|

- Bei der folgenden Art von Fragen entscheiden Sie sich bitte zwischen den Antwortvorgaben und kreuzen Sie das für Sie zutreffende an.

Beispiel: **Geschlecht:** ☒ Männlich ☐ Weiblich

- Bei einigen Fragen haben Sie die Möglichkeit, eine Antwort in eigenen Worten zu formulieren. Nutzen Sie bitte, die dafür vorgesehenen Linien und schreiben Sie nach Möglichkeit in Blockschrift oder Druckbuchstaben.

Beispiel:
In welchem Landkreis bzw. in welcher kreisfreien Stadt haben Sie die längste Zeit in Ihrem Leben gewohnt?
LANDKREIS OSNABRÜCK

- Falls Sie eine Antwort korrigieren möchten machen Sie dies bitte genau kenntlich.

Beispiel:

**16. Wie häufig unternehmen Sie in Ihrer Freizeit folgende Aktivitäten „im Grünen“?**				
	Nie	Selten (höchstens 1x/Monat)	Gelegentlich (mehrmals/pro Monat)	Häufig (min. 1x/Woche oder mehr)
spazieren gehen, wandern, joggen	☒ (durchgestrichen)	☒	☐	☐

Herzlichen Dank!
Ihr NLGA-Team

FRAGEBOGEN

Ausfülldatum des Fragebogens:

I__I__I.I__I__I.I__I__I__I__I

**ALLGEMEINE PERSONENANGABEN**	
**1.**	**Geschlecht:** ☐ Männlich ☐ Weiblich
**2.**	**Alter:** I__I__I Jahre
**3.**	**Postleitzahl Ihres aktuellen Wohnortes:** I__I__I__I__I__I
**4.**	**In welchem Landkreis bzw. in welcher kreisfreien Stadt haben Sie die längste Zeit in Ihrem Leben gewohnt?**<br>____
**ANGABEN ZUR WOHNLAGE**	
**5.**	**In welcher Wohnlage haben Sie die längste Zeit in Ihrem Leben gewohnt?**<br>☐ städtisch ☐ ländlich/ dörflich ☐ in Einzellage<br>Anders und zwar: ________________________________
**6.**	**Haben Sie jemals in der Nähe von … gewohnt?**<br>…einem Gewässer (z.B. Teich, See, Bach, Fluss, …)<br>☐ Nein ☐ Ja, bis zu 100 m ☐ Ja, mehr als 100 m ☐ Weiß nicht<br>…einem Waldrand oder kleineren Baumgruppen…<br>☐ Nein ☐ Ja, bis zu 100 m ☐ Ja, mehr als 100 m ☐ Weiß nicht<br>↳ Wenn ja, um was für eine Art von Bäumen handelt/e es sich?<br>☐ Nadelbäume ☐ Laubbäume ☐ Unbekannt<br>Falls genauer bekannt, bitte Baumart hier eintragen: ____________________

ANGABEN ZUR GESUNDHEIT

**7.**	**Wurde bei Ihnen jemals eine der folgenden chronischen Erkrankungen diagnostiziert?**				
	Diabetes	☐ Nein	☐ Ja →	In welchem Jahr oder Alter wurde die Diagnose gestellt?<br>Jahr |__|__|__|__| oder Alter |__|__|	☐ Weiß nicht
	Asthma/ COPD	☐ Nein	☐ Ja →	In welchem Jahr oder Alter wurde die Diagnose gestellt?<br>Jahr |__|__|__|__| oder Alter |__|__|	☐ Weiß nicht
	Multiple Sklerose	☐ Nein	☐ Ja →	In welchem Jahr oder Alter wurde die Diagnose gestellt?<br>Jahr |__|__|__|__| oder Alter |__|__|	☐ Weiß nicht
**8.**	**Nehmen Sie eines der folgenden, das Immunsystem unterdrückenden, Medikamente regelmäßig ein?**				
	Biologika[1]	☐ Nein	☐ Ja →	Seit wann?<br>Jahr |__|__|__|__| oder Alter |__|__|	☐ Weiß nicht
	Cortisonhaltige Medikamente	☐ Nein	☐ Ja →	Seit wann?<br>Jahr |__|__|__|__| oder Alter |__|__|	☐ Weiß nicht
**9.**	**Wurde bei Ihnen jemals eine *Infektion oder Erkrankung mit Hantaviren* festgestellt und wenn ja, wann?**				
		☐ Nein	☐ Ja →	In welchem Jahr oder Alter wurde die Diagnose gestellt?<br>Jahr |__|__|__|__| oder Alter |__|__|	☐ Weiß nicht
**10.**	**Wurde bei Ihnen jemals eine *Infektion mit Leptospiren/ Leptospirose* festgestellt und wenn ja, wann?**				
		☐ Nein	☐ Ja →	In welchem Jahr oder Alter wurde die Diagnose gestellt?<br>Jahr |__|__|__|__| oder Alter |__|__|	☐ Weiß nicht

[1] Biologika = bio-technologisch hergestellte Medikamente mit Wirkstoffen wie z.B. Adalimumab, Etanercept, Rituximab, Infliximab ,…

**11.**	**Hatten Sie jemals eines oder mehrere der folgenden Symptome, die im Rahmen einer akuten Erkrankung ohne eindeutige Ursache aufgetreten sind?**<br><br>Hirnhautentzündung ☐ Nein ☐ Ja ☐ Weiß nicht<br><br>Bindehautentzündung (‚rote Augen') ☐ Nein ☐ Ja ☐ Weiß nicht<br><br>Gelbsucht (‚gelbe Augen') ☐ Nein ☐ Ja ☐ Weiß nicht<br><br>Nierenfunktionsstörung/ Nierenversagen ☐ Nein ☐ Ja ☐ Weiß nicht
**12.**	**Rauchen Sie zurzeit – wenn auch nur gelegentlich?**<br><br>☐Ja → Wie viel rauchen Sie derzeit gewöhnlich?<br>I__I__I Anzahl Zigaretten pro Tag<br><br><u>Falls Sie regelmäßig[2] rauchen:</u><br>Wann haben Sie angefangen, regelmäßig[1] zu rauchen?<br>Jahr I__I__I__I__I oder Alter I__I__I<br><br>☐Nein, ich habe früher regelmäßig[2] geraucht, aber jetzt nicht mehr. → Wie viel haben Sie früher gewöhnlich geraucht?<br>I__I__I Anzahl Zigaretten pro Tag<br><br>Wann haben Sie angefangen regelmäßig[1] zu rauchen?<br>Jahr I__I__I__I__I oder Alter I__I__I<br><br>Wann haben Sie aufgehört zu rauchen?<br>Jahr I__I__I__I__I oder Alter I__I__I<br><br>☐Nein, ich habe noch <u>nie</u> regelmäßig[2] geraucht.

[2] <u>Unter regelmäßig verstehen wir:</u>
1 Zigarette pro Tag oder mindestens 5 Zigaretten pro Woche oder mindestens 1 Packung Zigaretten pro Monat für mindestens 6 Monate

**FREIZEITVERHALTEN**							
**13.**	**Wie viele Jahre Ihres Lebens hatten Sie oder ein Haushaltsmitglied eines oder mehrere der folgenden Haustiere?**						
		Nie	<1 Jahr	2-5 Jahre	6-10 Jahre	>10 Jahre	Ein Leben lang
	Hund	☐	☐	☐	☐	☐	☐
	Katze	☐	☐	☐	☐	☐	☐
	Reptil	☐	☐	☐	☐	☐	☐
	Nagetier -Welches? _______________	☐	☐	☐	☐	☐	☐
	Anderes Haustier - Welches? _______________	☐	☐	☐	☐	☐	☐

**14.**	**Wie lange gehen Sie durchschnittlich *unter der Woche (Mo-Fr)* in Ihrer Freizeit in der Zeit von Frühling bis Herbst folgenden Beschäftigungen nach?**<br>*Die Zeitangabe soll für alle Tage zusammen angegeben werden.*<br>*Die Angabe „2-5 Stunden“ bedeutet also 2-5 Stunden in der Woche von Mo-Fr.*					
		(Fast) gar nicht	<1 Stunde	2-5 Stunden	6-10 Stunden	>10 Stunden
	Aufenthalt im Freien	☐	☐	☐	☐	☐
	Aufenthalt im Wald	☐	☐	☐	☐	☐
	Arbeit mit Pflanzen und Erde, z.B. Gartenarbeit	☐	☐	☐	☐	☐
	Arbeit mit Holz, z.B. Sägen, Stapeln etc.	☐	☐	☐	☐	☐
	Kontakt zu Tieren	☐	☐	☐	☐	☐
	Arbeit mit Saatgut, Futtermittel, Dünger o.ä.	☐	☐	☐	☐	☐

15. **Wie lange gehen Sie durchschnittlich am *<u>Wochenende (Sa-So)</u>* in Ihrer Freizeit in der Zeit von Frühling bis Herbst folgenden Beschäftigungen nach?**
Die Zeitangabe soll für das gesamte Wochenende angegeben werden.
Die Angabe „2-5 Stunden" bedeutet also 2-5 Stunden an einem Wochenende (Sa-So).

	(Fast) gar nicht	<1 Stunde	2-5 Stunden	6-10 Stunden	>10 Stunden
Aufenthalt im Freien	☐	☐	☐	☐	☐
Aufenthalt im Wald	☐	☐	☐	☐	☐
Arbeit mit Pflanzen und Erde, z.B. Gartenarbeit	☐	☐	☐	☐	☐
Arbeit mit Holz, z.B. Sägen, Stapeln etc.	☐	☐	☐	☐	☐
Kontakt zu Tieren	☐	☐	☐	☐	☐
Arbeit mit Saatgut, Futtermittel, Dünger o.ä.	☐	☐	☐	☐	☐

16. **Wie häufig unternehmen Sie in Ihrer Freizeit folgende Aktivitäten „im Grünen"?**

	Nie	Selten (höchstens 1x/Monat)	Gelegentlich (mehrmals/ Monat)	Häufig (min. 1x/ Woche oder mehr)
Spazieren gehen, wandern, joggen	☐	☐	☐	☐
Jagen, Angeln *(in der jeweiligen Saison)*	☐	☐	☐	☐
Arbeit im Garten/ Schrebergarten	☐	☐	☐	☐
Sonstige Aktivitäten? - Welche? ________________________	☐	☐	☐	☐

17. **Wie häufig gehen Sie in Ihrer Freizeit folgenden Wassersportarten nach?**
(in der jeweiligen Saison)

	Nie	Selten (höchstens 1x/Monat)	Gelegentlich (mehrmals/ Monat)	Häufig (min. 1x/ Woche oder mehr)
Baden (z.B. im See, Bach oder Teich)	☐	☐	☐	☐
Segeln	☐	☐	☐	☐
Tauchen	☐	☐	☐	☐
Kanu/ Kajak/ Rafting	☐	☐	☐	☐
Surfen/ Kiten	☐	☐	☐	☐
Sonstige Wassersportarten? - Welche? ________________________	☐	☐	☐	☐

KONTAKT ZU NAGETIEREN

18. Wie häufig haben Sie in den vergangenen 10 Jahren in Ihrem Wohnumfeld/Arbeitsumfeld Anzeichen für Nagetiere entdeckt?

	Gar nicht	1-2 mal	3-5 mal	Mehr als 5 mal
Kot	☐	☐	☐	☐
Nagespuren	☐	☐	☐	☐
Schmierspuren	☐	☐	☐	☐
Nester, Bauten	☐	☐	☐	☐
Tote Tiere	☐	☐	☐	☐
Lebende Tiere	☐	☐	☐	☐
Veränderter Geruch	☐	☐	☐	☐

19. Wie häufig wurden bzw. haben Sie in den vergangenen 10 Jahren…?

	Gar nicht	1-2 mal	3-5 mal	Mehr als 5 mal
Von Nagetieren gebissen	☐	☐	☐	☐
Lebendige Nagetiere berührt	☐	☐	☐	☐
Tote Nagetiere berührt	☐	☐	☐	☐
Tierställe gereinigt	☐	☐	☐	☐
Lagerräume, Garagen, Dachböden o.ä. ausgefegt oder aufgeräumt	☐	☐	☐	☐

WISSEN ÜBER ERKRANKUNGEN

20. Haben Sie bereits von Hantaviren bzw. von Hantavirus-Erkrankungen gehört?

☐ Nein
→ weiter mit Frage 21

☐ Ja
Wenn Ja, wodurch und wie oft?
↓

	Nie	Ca. einmal	2-10 mal	> 10 mal	Immer wieder
Familienangehörige	☐	☐	☐	☐	☐
Bekannte, Kollegen, Nachbarn	☐	☐	☐	☐	☐
Lokalzeitung, Zeitschrift	☐	☐	☐	☐	☐
Fernsehen, Radio	☐	☐	☐	☐	☐
Internet, Soziale Medien (WhatsApp, Facebook)	☐	☐	☐	☐	☐
Information durch Gemeinde oder Landkreis	☐	☐	☐	☐	☐
Gesundheitsamt	☐	☐	☐	☐	☐
Information durch Arbeitgeber	☐	☐	☐	☐	☐

**21.**	**Haben Sie bereits von Leptospiren bzw. Leptospirose gehört?**<br>☐ Nein → weiter mit Frage 22<br>☐ Ja — Wenn Ja, wodurch und wie oft? ↓

	Nie	Ca. einmal	2-10 mal	> 10 mal	Immer wieder
Familienangehörige	☐	☐	☐	☐	☐
Bekannte, Kollegen, Nachbarn	☐	☐	☐	☐	☐
Lokalzeitung, Zeitschrift	☐	☐	☐	☐	☐
Fernsehen, Radio	☐	☐	☐	☐	☐
Internet, Soziale Medien (WhatsApp, Facebook)	☐	☐	☐	☐	☐
Information durch Gemeinde oder Landkreis	☐	☐	☐	☐	☐
Gesundheitsamt	☐	☐	☐	☐	☐
Information durch Arbeitgeber	☐	☐	☐	☐	☐

INFORMATIONEN IHRES GESUNDHEITSAMTES

**22a.**	**Sind Ihnen die Pressemitteilungen Ihres Gesundheitsamtes zu Hantavirus-Erkrankungen/ Leptospirose bekannt?**<br>☐ Nein → weiter mit Frage 23<br>☐ Ja
**22b.**	**Waren die Pressemitteilungen des Gesundheitsamtes hilfreich für Sie?**<br>☐ Nein ☐ Ja ☐ Nicht gelesen
**22c.**	**Wo haben Sie die Pressemitteilung des Gesundheitsamtes gesehen/gelesen?**<br>☐ Zeitung ☐ Internet<br>Anderswo? → Wenn ja, wo?<br>Hier bitte eintragen: ___________________________
**22d.**	**Über welches Medium würden Sie von Ihrem Gesundheitsamt zukünftig gern informiert werden?**<br>☐ Beim Arzt ☐ Per Zeitung ☐ Per Post<br>☐ Per E-Mail ☐ über Soziale Medien (z.B. Facebook, Twitter,…) ☐ Radio<br>☐ Fernsehen ☐ Gar nicht<br>Anders? → Wenn ja, wie?<br>Hier bitte eintragen: ___________________________

**Anwendung von Präventionsmaßnahmen**					
**23a.**	**Haben Sie in der Vergangenheit bewusst Maßnahmen zum Schutz vor nagetierübertragenen Infektionen ergriffen?**				
		Nie	Manchmal	Immer	Trifft nicht zu
	Tragen eines **Mund- und Nasenschutzes** z.B. beim Ausfegen von Lagerräumen	☐	☐	☐	☐
	Tragen von **Schutzhandschuhen** z.B. beim Berühren von Nagetieren	☐	☐	☐	☐
	**Händewaschen** nach dem Berühren von Nagetieren	☐	☐	☐	☐
**23b.**	**Haben Sie in der Vergangenheit bewusst Maßnahmen ergriffen um Ihr zu Hause frei von Mäusen zu halten?**				
		Nie	Manchmal	Immer	Trifft nicht zu
	Abfallbeseitigung in verschließbaren Müllbeuteln	☐	☐	☐	☐
	Mögliche Eintrittsstellen ins Haus abgedichtet	☐	☐	☐	☐
	Unterschlupf- und Nistmöglichkeiten für Nager beseitigt	☐	☐	☐	☐
**23c.**	**Haben Sie in der Vergangenheit bewusst Maßnahmen zur Bekämpfung von Mäusen ergriffen?**				
		Nie	Manchmal	Immer	Trifft nicht zu
	Schlagfallen	☐	☐	☐	☐
	Nagergifte (Rodentizide)	☐	☐	☐	☐
	Schädlingsbekämpfer	☐	☐	☐	☐
**23d.**	**Haben Sie in der Vergangenheit bewusst folgende Maßnahmen bei der Beseitigung von Mäusen, Mäuseausscheidungen und der abschließenden Reinigung beachtet?**				
		Nie	Manchmal	Immer	Trifft nicht zu
	Lüften vor der Reinigung eines Raumes mit Mausbefall	☐	☐	☐	☐
	Staubaufwirbelung vermeiden bei der Entfernung von Mäusekot und Nestmaterial	☐	☐	☐	☐
	Reinigung aller gebrauchten Fallen	☐	☐	☐	☐

SCHULBILDUNG UND BERUF

24. **Welchen höchsten allgemeinbildenden Schulabschluss haben Sie?**

- Schüler/in, Besuch einer allgemeinbildenden Vollzeitschule ☐
- Von der Schule abgegangen ohne Schulabschluss ☐
- Hauptschulabschluss (Volksschulabschluss) oder gleichwertiger Abschluss ☐
- Realschulabschluss (Mittlere Reife) oder gleichwertiger Abschluss ☐
- Abitur/ Allgemeine oder fachgebundene Hochschulreife (Gymnasium bzw. EOS, auch EOS mit Lehre) ☐
- Einen anderen Schulabschluss? - Welchen?

____

25. **Sind Sie jemals haupt- oder nebenberuflich tätig gewesen, …?**
(Mehrfachnennungen sind möglich)

Bitte Jahreszahlen eintragen.

	Nein	Ja	Von…	Bis…
…als Landwirt/ Schäfer	☐	☐ →	I__I__I__I__I	I__I__I__I__I
…als Gärtner bzw. im Gartenbau/ Straßenmeisterei	☐	☐ →	I__I__I__I__I	I__I__I__I__I
…als Förster/ Forstbediensteter	☐	☐ →	I__I__I__I__I	I__I__I__I__I
Als Arbeiter/In in der Abfallwirtschaft (z.B. Müllentsorgung, Kanal/Abwasser, …)	☐	☐ →	I__I__I__I__I	I__I__I__I__I
…als Jäger	☐	☐ →	I__I__I__I__I	I__I__I__I__I
…in einem anderem Beruf, in dem Sie sich viel in der freien Natur aufgehalten haben?	☐	☐ →	I__I__I__I__I	I__I__I__I__I

Wenn ja, bitte **Berufsbezeichnung** hier eintragen.
↓

____

Vielen Dank!
